# Isaac Douglas, M.D., D.D..S

**Published:** 1910-03-15

**Authors:** 


### OBITUARY.

---

Isaac Douglas, M.D., D.D..S, Dr. Douglas was probably the oldest practitioner of dentistry in the State of Michigan at the time of his death. He was a consistant member and attendant of the State and other societies for many years. He died at Romeo, Mich., October 14th, 1909, at the age of 79 years. He began the study of dentistry with his brothei at Romeo in 1852. He was a graduate of the Ohio Dental College and the Cleveland Homeopathic Medical College.

